# Control of HSV-1 Infection: Directions for the Development of CRISPR/Cas-Based Therapeutics and Diagnostics

**DOI:** 10.3390/ijms252212346

**Published:** 2024-11-17

**Authors:** Anastasiia O. Sosnovtseva, Natalia A. Demidova, Regina R. Klimova, Maxim A. Kovalev, Alla A. Kushch, Elizaveta S. Starodubova, Anastasia A. Latanova, Dmitry S. Karpov

**Affiliations:** 1 Engelhardt Institute of Molecular Biology, Russian Academy of Sciences, Vavilov Str., 32, 119991 Moscow, Russia; sollomia@yandex.ru (A.O.S.); kovalev_maksim_2002@mail.ru (M.A.K.); estarodubova@yandex.ru (E.S.S.); aalatanova@gmail.com (A.A.L.); 2 Center for Precision Genome Editing and Genetic Technologies for Biomedicine, Engelhardt Institute of Molecular Biology, Russian Academy of Sciences, Vavilov Str., 32, 119991 Moscow, Russia; 3 N.F. Gamaleya National Research Centre for Epidemiology and Microbiology, Ministry of Health of the Russian Federation, Gamaleya Str., 18, 123098 Moscow, Russia; ailande@yandex.ru (N.A.D.); regi.k@mail.ru (R.R.K.); vitallku@mail.ru (A.A.K.)

**Keywords:** CRISPR/Cas9, CRISPR/CasX, herpes simplex virus type 1, latent infection

## Abstract

It is estimated that nearly all individuals have been infected with herpesviruses, with herpes simplex virus type 1 (HSV-1) representing the most prevalent virus. In most cases, HSV-1 causes non-life-threatening skin damage in adults. However, in patients with compromised immune systems, it can cause serious diseases, including death. The situation is further complicated by the emergence of strains that are resistant to both traditional and novel antiviral drugs. It is, therefore, imperative that new methods of combating HSV-1 and other herpesviruses be developed without delay. CRISPR/Cas systems may prove an effective means of controlling herpesvirus infections. This review presents the current understanding of the underlying molecular mechanisms of HSV-1 infection and discusses four potential applications of CRISPR/Cas systems in the fight against HSV-1 infections. These include the search for viral and cellular genes that may serve as effective targets, the optimization of anti-HSV-1 activity of CRISPR/Cas systems in vivo, the development of CRISPR/Cas-based HSV-1 diagnostics, and the validation of HSV-1 drug resistance mutations.

## 1. Introduction

It is estimated that over 90% of the global population is infected with herpesviruses. Herpes simplex virus type 1 (HSV-1) is one of the most prevalent herpesviruses and establishes a lifelong latent infection in sensory neurons [1]. HSV-1 most often replicates in the epithelium and, in the majority of cases, causes non-serious lesions. However, it can also lead to more serious disease, including recurrent keratitis, which can result in blindness [2], herpes simplex encephalitis, which can cause death, and residual neurological disorders in survivors [3]. Additionally, HSV-1 can cause systemic neonatal infection, which is one of the deadliest neonatal infections, with a mortality rate of 40% [4]. HSV-1, as with other members of the Alphaherpesvirinae family, is distinguished by a brief reproductive cycle, rapid destruction of host cells, and the capacity to establish a latent infection in the sensory ganglia. HSV-1 represents a significant threat to individuals with compromised immune systems [5]. Additionally, there is evidence to suggest that HSV-1 infection may be associated with the development of Alzheimer’s disease [6]. The primary therapeutic approach for HSV-1 is the utilization of nucleoside analogs, such as acyclovir (ACV). These pharmaceutical agents are capable of suppressing active infection but are ineffective in combating latent infection [7]. Furthermore, immunocompromised patients frequently develop strains that are resistant to antiviral therapy as a result of mutations in the viral thymidine kinase (TK) and DNA polymerase [8]. A novel class of drugs for HSV-1 therapy comprises helicase–primase inhibitors, which exhibit enhanced efficacy against active lytic infection and the capacity to inhibit latent forms of HSV-1 [9,10]. However, there have been reports of the emergence of HSV-1 forms resistant to helicase–primase inhibitors [11], indicating an urgent need for the development of new drugs and approaches to HSV-1 therapy. Currently, clustered, regularly interspaced short palindromic repeat (CRISPR/Cas) systems are being actively investigated as a potential therapy for various diseases, including genetic diseases, autoimmune diseases, cancers, and viral diseases.

CRISPR/Cas systems serve as an adaptive immune response in bacteria, archaea, and giant viruses against foreign nucleic acids, most commonly bacteriophages [12,13]. Natural CRISPR/Cas systems can be classified into two classes, each comprising three distinct types. The class 1 encompasses systems comprising multiple Cas proteins, which co-assemble to form a multi-protein complex. This class includes types I, III, and IV. Given their intricate structure, they are more frequently employed in host organisms for genome editing purposes. Class 2 encompasses types II, V, and VI, in which the functions of target recognition and cleavage are performed by a single multidomain protein. The protein utilizes the guide RNA as a single molecule (in types V and VI) or as a complex of two gRNA and tracrRNA molecules (type II) depending on the type of CRISPR/Cas system [14]. 

The *S. pyogenes* type II-A CRISPR/Cas9 system was the first to be employed in the field of genome editing, a technology that has become widely utilized in a range of research and applications, including human gene therapy [15]. Cas9 from *S. pyogenes* is the most extensively studied and widely utilized genomic and post-genomic editor. This is likely due to the fact that Cas9 is generally more active in eukaryotic cells than other Cas effectors, as evidenced by both in vitro [16] and in vivo [17] studies. However, *S. pyogenes* Cas9 has several disadvantages, including its large size and relatively high level of off-targets [18]. The large size of Cas9 limits its packaging into AAV vectors approved by the FDA for gene delivery [19]. Furthermore, the high level of off-targeting raises concerns about the safety of Cas9-based therapies. Consequently, Cas9 orthologs and Cas effectors of other types with smaller sizes and relatively high specificity are being studied for use in the treatment of human genetic diseases and viral infections [20,21].

The objective of this review is to examine the current advancements in four principal areas of CRISPR/Cas system development as anti-HSV-1 therapeutic agents: (1) the identification of viral and cellular genes as effective targets for CRISPR/Cas systems, (2) the development and improvement of anti-HSV-1 therapy based on CRISPR/Cas systems in vivo, (3) CRISPR/Cas-based HSV-1 detection, and (4) CRISPR/Cas-based validation of drug-resistant HSV-1 mutations. 

## 2. Molecular Mechanisms of HSV-1 Infection: The Basis for CRISPR/Cas9 Targets Selection

Prior to an examination of the prospective avenues for the advancement of CRISPR/Cas-based therapies for HSV-1 infection, it is essential to gain an understanding of the prevailing knowledge concerning the molecular mechanisms and pivotal genes that regulate the life cycle of this virus. In Section 3, we will discuss which of these viral and cellular genes can be considered as targets for CRIPSR/Cas9 editing. 

HSV-1 is a member of the Herpesviridae family, specifically the Alphaherpesvirinae subfamily and the *Simplexvirus* genus. It is a neurotropic virus, yet the primary infection typically occurs in the mucosal epithelium. Subsequently, the virus disseminates via retrograde axonal transport to the nuclei of peripheral sensory neurons, where it may establish a latent infection [22]. In the absence of an effective host response to HSV-1 infection, the virus is capable of disseminating throughout the organism, a phenomenon that is most frequently observed in infants or immunocompromised adults [5,23]. In rare instances, primary HSV-1 infection and reactivation from a latent state can result in HSV-1 invasion of the central nervous system, with significant consequences, including blindness, hearing loss, and fatal encephalitis [24]. 

The HSV-1 genome is about 152 kb in length and is composed of double-stranded DNA, which encodes at least 84 open reading frames (ORFs), a non-coding RNA known as the latency-associated transcript (LAT), and several microRNAs (miRNAs) [25]. The genome is organized as TRL-UL-IRL-IRS-US-TRS, with two regions of unique sequences, unique long (UL) and unique short (US), flanked by two sets of inverted repeats, TRL/IRL (terminal repeat long/internal repeat long), and TRS/IRS (terminal repeat short/internal repeat short) [26]. As a consequence of this genomic architecture, the UL and US regions may undergo inversion during the course of DNA replication. The genes that are encoded by the UL and US regions are designated as UL and US genes, respectively. 

**Figure 1:**
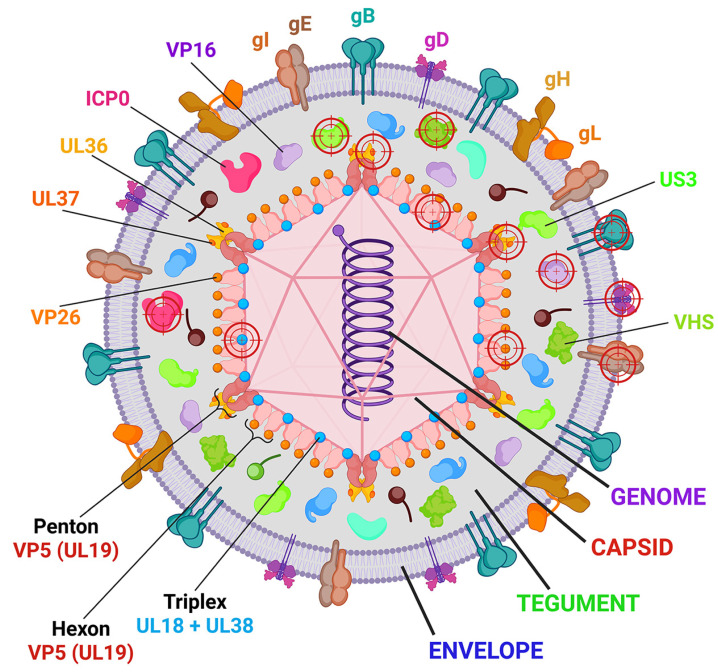
The schematic structure of the HSV-1 particle with targets of the CRISPR/Cas system. The genome of HSV-1 is represented by double-stranded DNA, which is packaged into an icosahedral capsid. The primary structural protein of the capsid is VP5 (UL19), which forms hexon (on the facets) and penton (on the angles) complexes. The tegument, a protein shell located on the exterior of the capsid, contains several viral proteins, including VP16, ICP0, US3, VHS, and others. The outermost side of the virion is the envelope, which is a membrane that contains embedded glycoproteins, including gI, gE, gB, gD, gH, and gL. The red target symbols indicate viral proteins whose genes are the intended targets of CRISPR/Cas-based therapies. Abbreviations: g (in gI, gE, gB, gD, gH, gL)—glycoprotein; US3—unique short region 3; VHS—virion host shutoff; UL (in UL8, UL38, UL19, UL37, UL36)—unique long (region); VP (in VP5, VP26, VP16)—viral protein; ICP0—infected cell protein 0.

The HSV-1 particle has a diameter of approximately 200 nm and is composed of an icosahedral capsid encased in an amorphous proteinaceous coat, the tegument, which in turn is surrounded by an envelope (Figure 1). The capsid encapsulates the viral genome and is constructed from the products of approximately 50–60 HSV-1 genes, which represent approximately 75% of the genome. The major capsid protein, VP5 (*UL19* gene), is responsible for forming the major capsid units, hexons and pentons [27] (Figure 1). The tegument comprises over 20 structural and regulatory proteins, in addition to viral RNAs and cellular components. Some of the tegument proteins are covalently linked to capsid proteins and are, therefore, arranged in the same icosahedral pattern as the capsid proteins [28]. A lipid bilayer envelope contains at least 12 different viral glycoproteins, the majority of which are monomers, with some being heterodimers (gH/gL and gE/gI). 

**Figure 2:**
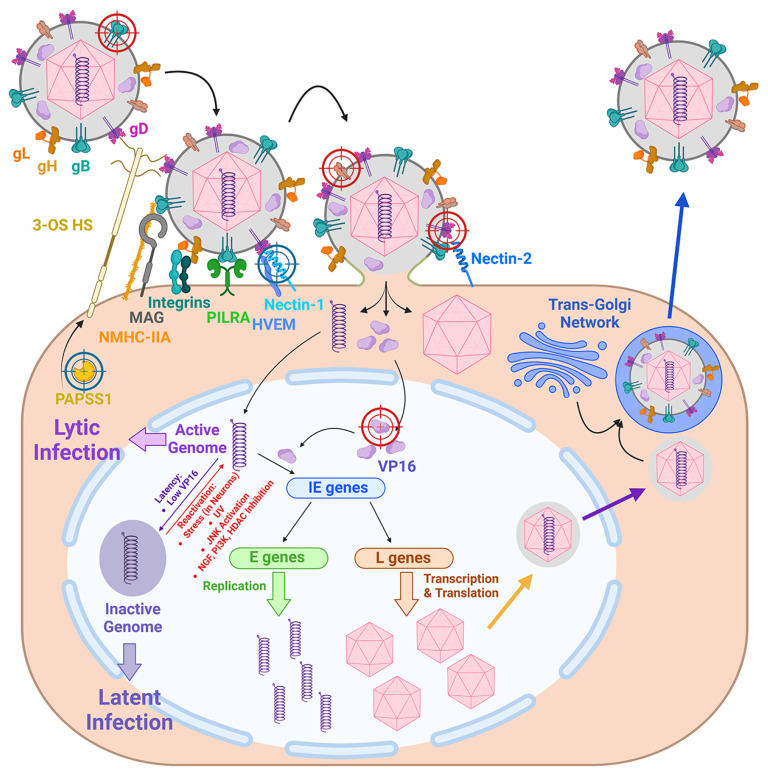
The general scheme of the HSV-1 life cycle with targets of the CRISPR/Cas system. Infection commences with the interaction of HSV-1 envelope glycoproteins with receptors on the cell membrane. These include gD with 3-OS HS (synthesized by PAPSS1), HVEM, and nectin-1,2; gH/gL with integrins; and gB with NMHC-IIA, MAG, and PILRA. Subsequently, the virus gains entry into the cell, with the viral genome and the VP16 protein moving into the nucleus. In the event that VP16 levels are insufficient, the genome may be inactivated, resulting in a latent infection. Conversely, reactivation may occur as a consequence of stress, ultraviolet radiation, JNK activation, and inhibition of the NGF, PI3K, and HDAC pathways, leading to a lytic infection. Subsequently, VP16 activates the IE genes, which in turn induce the expression of the E (essential for genome replication) and L (necessary for genome transcription and synthesis of capsid and tegument proteins) genes. Subsequently, the capsids are assembled and subsequently exit the nucleus. The assembly of viral particles is completed in the cytoplasm and the trans-Golgi network, after which the particles leave the cell. Red target symbols indicate viral proteins whose genes are targets of CRISPR/Cas-based therapy, while blue target symbols represent analogous host cell proteins. The arrows show the successive stages of the virus life cycle. Abbreviations: g (in gB, gD, gH, gL)—glycoprotein; 3-OS HS—3-O-sulfated heparan sulfate; PAPSS1—3-phosphoadenosine 5′-phosphosulfate synthetase 1; NMHC-IIA—non-muscle myosin heavy chain IIA; MAG—myelin-associated glycoprotein; PILRA—Paired immunoglobulin-like type 2 receptor α; HVEM—herpesvirus entry mediator; VP16—viral protein 16; IE (genes)—immediately early; E (genes)—early; L (genes)—late; UV—ultraviolet; JNK—c-Jun N-terminal kinase; NGF—nerve growth factor; PI3K—phosphoinositide 3-kinases; HDAC—histone deacetylase.

HSV-1 glycoproteins bind to numerous host cell surface proteins, including the paired immunoglobulin-like type 2 receptor alpha (PILRa) [29], myelin-associated glycoprotein (MAG) [30], nonmuscle myosin heavy chain IIA and IIB (NMHC-IIA/IIB) [31], integrins αvβ6 and αvβ8 [32], herpesvirus entry mediator (HVEM), PILRa, NECTIN-1, NECTIN-2, and 3-O-sulfated heparan sulfate proteoglycan (3-OS-HS) [33] (Figure 2). This binding process is followed by the interaction of viral glycoproteins with each other, subsequently leading to the fusion of the viral envelope with the cell membrane [34] and resulting in the formation of a fusion pore, which allows the virus to release the capsid and the specific subset of the inner tegument proteins into the cytoplasm of the host cell [33]. They are transported in the retrograde direction to the nucleus along microtubules, and upon reaching it, the capsid is uncoated, and the viral DNA is released from it through the nuclear pore to the nucleus in a linear form [35]. Upon fusion of the virus with the cellular membrane, a portion of the tegument proteins detach from the capsid and can either independently translocate to the nucleus as the transcriptional activator VP16 [36] or remain in the cytoplasm as the VHS protein (*UL41* gene). VHS is an endoribonuclease that cleaves both cellular and viral mRNAs with low specificity [37], resulting in significant inhibition of host protein synthesis and thereby facilitating the transcription of viral genes at subsequent stages of the viral cycle [38].

In the nucleus, the viral genome is rapidly circularized by cellular DNA ligase IV, which joins the non-homologous DNA ends during a cellular repair process [39]. In sensory neurons, viral DNA more likely will persist in an episomal form for decades, thereby establishing a latent infection. In epithelial cells, however, the likelihood of a lytic infection cycle is greater [25]. In the latter case, viral transcription is initiated as an ordered cascade, commencing with the expression of immediate early (IE) genes, followed by the expression of early (E) and then late (L) genes [40]. Transcription is regulated by the protein products of the viral IE and E genes, as well as by host proteins, including host RNA polymerase II [41]. Of note, at the very beginning of the HSV-1 cycle, viral proteins ICP4 and ICP0 prevent initiation of the transcription of all viral genes [42] by binding to a specific motif of IE gene promoters and by impeding the recruitment of RNA polymerase II to promoters [43,44]. Later, viral protein VP16, delivered to the nucleus upon viral entry, promptly reverses repression on IE genes [45] by binding to their specific promoter sequence, TAATGARAT, in association with the cellular host proteins Oct-1 and HCF [46]. The mRNAs of transcribed IE genes are transported to the cytoplasm and translated into five IE proteins, including ICP4, ICP0, ICP27, and ICP22, which are transported back to the nucleus. They mediate E and then L gene transcription [44,47,48,49,50]. 

As a result of E gene expression, the proteins of the viral replication machinery are synthesized, including a two-subunit viral DNA polymerase (UL30, a catalytic subunit, and UL42, processivity factor), the origin-binding protein (UL9), the heterotrimeric helicase–primase complex (UL5, UL8, UL52), and the single-stranded DNA-binding protein (UL29, or ICP8) [25,51]. This is followed by HSV-1 genome replication, which is thought to start as origin-dependent/theta replication at one of the three viral origins of replication [52] and is later converted to a rolling-circle form that yields long concatemeric DNA [53]. The UL9 protein cooperatively binds to specific sequences of the Ori, resulting in distortion of the A/T-rich spacer region [54]. It also binds to the ICP8 protein, which stimulates UL9 ATP-dependent helicase activity, resulting in DNA unwinding in the 3′ -> 5′ direction [55,56]. ICP8 also binds to the unwinded single-stranded DNA [57], resulting in the formation of high-order filaments and double-helical structures, which are postulated to function as a protein scaffold that may facilitate the concentration of viral DNA and viral and cellular proteins essential for replication [58]. Subsequently, the helicase/primase complex is recruited to unwind duplex DNA (UL5 helicase activity) and to synthesize short RNA primers, serving as substrates for viral DNA synthesis (UL52 primase activity). UL8, an accessory factor, is responsible for ensuring the nuclear localization of the entire complex and also contributes to dsDNA unwinding [59]. Ultimately, the polymerase UL30/UL42 complex is recruited to the replication fork, where it utilizes RNA primers to facilitate the synthesis of both the leading and lagging viral DNA strands. It results in the formation of head-to-tail concatemers, which are composed of multiple copies of the viral genome. Specific sites of the HSV-1 genomic termini undergo cleavage, which results in the formation of unit-length monomers that are then packaged into procapsids [60].

The efficiency of replication may be enhanced by accessory enzymes encoded by the other HSV-1 E genes: TK (*UL23*, or *ICP36*) and ribonucleotide reductase (*UL39*/*UL40*) catalyze the synthesis of dNTP substrates and favor HSV-1 replication in neurons [61,62]; deoxyuridine triphosphatase (*UL50*) is essential for the synthesis of thymidine and maintains a low pool of dUTP, which may help to prevent dUTP incorporation into the replicating viral genomes [63]; uracil DNA glycosylase (*UL2*) removes uracil from DNA; and alkaline nuclease (*UL12*) has 5′-3′ exonuclease activity [64,65]. 

It has been proposed that the replication process of HSV-1 is dependent on recombination, as is the case for phage lambda [66,67]. Recombination is an important driver of genetic variation and evolution of HSV-1, as its genetic mutation rates are low due to the highly efficient proof-reading and exonuclease activity of its DNA polymerase [68]. A two-component HSV-1 recombinase, UL12/ICP8, has been demonstrated to perform recombination during replication by promoting strand annealing reactions in conjunction with host factors [66,69,70]. 

The replication of viral DNA, in conjunction with IE and E protein production, is necessary for the transcription of L genes [51]. Specific mechanisms play a pivotal role in the expression of L genes, i.e., the VHS protein causes the nuclear retention of viral RNAs from the IE and E genes while allowing for the efficient transport of L transcripts to the cytoplasm [71,72]. As a result of L gene transcription, viral structural proteins and those required for viral assembly are produced [73]. Proteins of the viral outer tegument and envelope are incorporated into the membranes of the trans-Golgi network and endosomes [74]. Subsequent processing of the precursor glycoproteins, including glycosylation, occurs in the endoplasmic reticulum (ER) and the Golgi apparatus. Other L proteins are transported to the nucleus, where they are essential for the assembly of capsids and for cleaving concatemeric DNA, enabling the simultaneous release and packaging of genomes [40]. 

The maturation of the capsid for genome encapsidation is initiated by the viral protease VP24 [75], while cellular proteins such as importin α facilitate the efficient import of newly synthesized viral proteins from the cytoplasm to the nucleus and are involved in capsid assembly and egress [76]. The mature capsids, carrying the viral DNA, receive their primary envelope as they bud into the inner nuclear membrane and pinch off from it, which is mediated by the nuclear exit complex consisting of viral (i.e., UL31, UL34) and cellular (i.e., lamin A/C) proteins [40,77]. Subsequently, the primary viral envelope fuses with the outer nuclear membrane, resulting in the release of the capsid into the cytoplasm. Prior to or shortly after its egress from the nucleus, the capsid becomes associated with inner tegument proteins, including UL36 and UL37, as well as US3, a serine/threonine kinase. These proteins play a role in capsid recruitment to the inner nuclear membrane and facilitate its delivery to the trans-Golgi network/endosome compartment [3,78]. In addition, UL36 facilitates the recruitment of outer tegument proteins, including VP16, which in turn attracts VP11/2, VP13/14, VP22, and membrane-embedded envelope proteins [79]. The capsids bud into the membranes of the trans-Golgi network/endosomes and acquire envelopes with mature viral membrane proteins and the outer tegument proteins. Furthermore, axonal transport of fully assembled viral particles in a direction from the cell body to the ends of the axon is enabled by the viral gD glycoprotein [80]. Cellular vesicles transport HSV-1 particles to the plasma membrane, and the viral particles are released when the vesicles fuse with the membrane [3].

Within a period of one to two weeks after acute infection of the peripheral ganglia, HSV-1 may establish latent infection, which is induced by heterochromatinization of the viral genome. Upon entering the cell, viral DNA interacts with pattern recognition receptors (i.e., IFI16) [81] or with host nuclear structures (promyelocytic leukemia nuclear bodies) [82], which exposes DNA to histones and histone modifications by various enzymes. As a result, the lytic genes are silenced, and the HSV-1 genome persists within the nucleus in a non-integrated form as a circular episome [83,84]. However, in latently infected neurons, one transcriptionally active locus, LAT (latency-associated transcript), is detected [85,86]. Its primary transcript, measuring 8.3–8.9 kb, is unstable; however, rapid splicing results in the production of two stable introns, measuring 2.0 and 1.5 kb, respectively [87]. The former is regarded as the hallmark of HSV-1 latency [88]. It is hypothesized that LAT can influence the cellular histone modification apparatus [89] and that miRNAs encoded by the LAT region may inhibit the expression of lytic viral genes [90]. LAT also interacts with the immune system, as evidenced by its transcription, delaying the production of some IFN-α and IFN-β, which may be related to HSV-1 latency [91].

The establishment of a latent HSV-1 infection provides a viral reservoir for periodic reactivation. The most evident mechanism is that reactivation is prompted by alterations in the chromatin state induced by a variety of stimuli. In humans, these include exposure to UV radiation, an altered hormonal background, fever, or trauma [92,93]; in cell culture and animal studies, neuronal stress, exposure to UV radiation, inhibition of phosphoinositide 3-kinase (PI3K), inhibition of histone deacetylases, and activation of the N-terminal c-Jun kinase JNK pathway [93]. As a result of reactivation, HSV-1 genes are expressed, the genomes are replicated, and productive progeny viruses are formed [94,95,96]. The newly assembled virions then move along the axon in an anterograde direction, reaching the initial or new site in the skin or mucosa, where the next round of replication can take place [97]. 

## 3. Directions for the Use of CRISPR/Cas Systems in the Control of HSV-1 Infections

### 3.1. The First Direction: The Search for Cellular and Viral Genes as Effective Targets for CRISPR/Cas Systems

A multitude of cellular proteins are implicated in the HSV-1 life cycle, exerting either inhibitory or stimulatory effects on the infectious process. The identification and characterization of host factors involved in the virus life cycle can provide crucial insights into the intricacies of virus–host relationships, ultimately leading to the discovery of novel, effective antiviral targets. The advent of CRISPR-Cas-based gene editing technologies has facilitated the identification of host factors indispensable for virus replication. CRISPR-Cas methodologies have yielded intriguing insights not only for HSV-1 but also for other significant viral pathogens, including dengue virus, Zika virus, West Nile virus, and hepatitis C virus [98].

In comparison to preceding genetic technologies, CRISPR/Cas9 provides a novel and robust methodology for the identification of host genes indispensable for HSV-1 infection through the utilization of a library of sgRNAs targeting a vast majority of known host genes in a knockout screen. Thus, Suzuki T et al. were able to identify a set of genes involved in heparan sulfate (HepS) biosynthesis as host factors that play an essential role in the process of HSV-1 cell entry [99]. In this discovery, the authors identified 3′-phosphoadenosine-5′-phosphosulfate synthase 1 (PAPSS1) as a pivotal enzyme in HepS biosynthesis. The knockout of PAPSS1 has been demonstrated to reduce HepS expression, thereby inhibiting HSV-1 binding to the cell surface [99]. Johnson K.E. et al. [100] examined the impact of the interferon-inducible protein 16 (IFI16) on HSV-1 replication in human cells. IFI16 is a multifunctional nuclear protein that plays a role in sensing viral DNA (including that of HSV-1) [100] and RNA [101], as well as in the induction of interferon-beta expression and the activation of the inflammasome [102]. Following the invasion of the viral DNA into the nucleus, IFI16 relocates from the nucleolus to the nuclear periphery, where it binds to the HSV-1 DNA and modulates viral and cellular transcription through mechanisms that remain largely undetermined. The knockdown of the *IFI16* gene via CRISPR/Cas9 resulted in a sixfold increase in HSV-1 yield. In contrast, overexpression of *IFI16* resulted in a reduction in virus yield by more than fivefold [100]. IFI16 exhibits a preferential binding affinity for the *UL30* and *US1-US7* genes [103]. The *UL30* and *US1* gene products are indispensable for HSV-1 replication. Another host factor, SMCHD1, acting as a suppressor for HSV-1 infection, has been identified via CRISPR/Cas9 screening [104]. SMCHD1 is a component of the cell-intrinsic antiviral immunity system and restricts the replication of herpesviruses by binding the origins of viral DNA replication. Moreover, using CRISPR/Cas9 screening, the authors confirmed other host genes that restrict HSV-1 replication, e.g., IRF8 [104].

In a study published by Turner E.M. et al. [105], the CRISPR/Cas9 system was employed to investigate the function of the Torsin/cofactor system in the context of HSV-1 infection. Torsins are the sole AAA+ ATPases that reside within the nuclear envelope and endoplasmic reticulum network. Torsin activity is dependent on two cofactors, namely transmembrane polypeptides, lamin-associated polypeptide 1 (LAP1), and luminal domain-like LAP1 (LULL1) [106]. The knockout of LULL1 via CRISPR/Cas9 reduces HSV-1 growth by approximately one order of magnitude, while the deletion of other components of the torsin system results in subtle defects [105]. LULL1 deficiency results in a 10-fold reduction in the number of viral genomes per host cell without affecting viral protein production. The authors have demonstrated that the assembly and packaging of viral capsids in the nucleus is significantly delayed in LULL1 knockout cells. This indicates that the activity of LULL1 is essential for capsid assembly and HSV-1 maturation at a step preceding nuclear egress.

In a recent study, Li et al. employed the CRISPR/Cas9 system to inhibit the function of one of the HSV-1 receptors on human corneal epithelial cells (HCECs), namely Nectin cell adhesion molecule 1 (NECTIN-1) [107]. It has previously been demonstrated that the HSV-1 envelope protein gD binds to NECTIN-1, facilitating virus penetration into human epithelial cells and neurons ex vivo [108]. HSV-1 infection of HCECs results in herpetic stromal keratitis (HSK), a lifelong, recurrent disease that has the potential to cause blindness. The current antiviral therapy is unable to eliminate the transcription-silent HSV-1 in latently infected patients. To establish two NECTIN-1 knockdown cell lines, the authors used lentiviral particles for the delivery of CRISPR/Cas9 components targeting human NECTIN-1. The knockdown of NECTIN-1 resulted in a significant reduction in HSV-1 DNA content and infection rate. These findings demonstrate the potential of CRISPR/Cas9 in the treatment of HSK. 

Consequently, CRISPR/Cas9 screens offer a novel and efficacious approach to identifying pivotal host factors that either promote or inhibit HSV-1 infection. Factors that stimulate virus infection could be regarded as prospective therapeutic targets.

Key viral proteins involved in the viral life cycle, latency, and reactivation may also represent important targets for antiviral therapy. CRISPR/Cas-mediated screening may be employed for the identification of HSV-1 genes that are crucial for infection; a protocol for this approach has recently been published [109]. Nevertheless, no published work has yet addressed the use of CRISPR screening in the search for essential HSV-1 genes. The available work has focused on targeting the CRISPR/Cas systems against single genes, as well as two, three, or four genes simultaneously. Targeting multiple genes simultaneously represents another advantage and a novel approach to searching for important viral genes using the CRISPR/Cas systems. At present, 29 HSV-1 genes have been targeted by CRISPR/Cas systems, and the impact of their inactivation on HSV-1 infection has been studied (see Table 1). The majority of viral gene knockouts resulted in a restricted viral infection, as evidenced by a reduction in replication [110,111,112,113,114,115,116,117,118], viral particle production [110,111,113,114,115,119,120], viral spreading [115,117], or providing protection against viral infection [110] in in vitro systems. Additionally, the inhibition of HSV-1 replication and reactivation has been demonstrated in an in vivo rabbit keratitis model [121]. It is noteworthy that the introduction of vectors with the CRISPR/Cas9 system, in the absence of sgRNA (single-guide RNA) or non-target sgRNA, has also been observed to result in a transient attenuation of infection. This observation has been documented in multiple studies, as referenced in [112,113,114]. This phenomenon may be attributed to the activation of cellular defense mechanisms in response to the expression of components of the CRISPR/Cas9 system or the non-specific activity of the Cas9 protein itself. The targeting of the CRISPR/Cas9 system to almost any HSV-1 gene [111,112,113,120], including non-essential genes [112,120], led to the attenuation of infection. However, the severity and duration of the effect differed. In a study conducted by Van Diemen and colleagues, the administration of sgRNA sequences targeting seven distinct HSV-1 genes (*UL8*, *UL 29*, *UL 36*, *UL52*, *UL 54*, *US3*, *US8*), resulted in impaired virus replication on day 2 post-infection. However, this effect was either reduced or completely reversed on day 3, particularly in the case of the *UL36*, *UL54*, *US3*, and *US8* genes [112]. The simultaneous use of multiple anti-HSV-1 sgRNAs has been demonstrated to effectively suppress infection in several studies [111,113,114]. Ying et al. assessed the antiviral impact of CRISPR/Cas9 targeting four distinct HSV-1 genes (*VP16*, *ICP27*, *ICP4*, or *US6*) and their collective effect. The authors demonstrated that targeting Cas9 to each of these genes resulted in a notable inhibition of HSV-1 infection and replication in vitro. However, this effect was found to be more pronounced when the genes were employed in a simultaneous manner [111]. Similarly, other studies have also documented an enhanced antiviral effect when targeting dual or multiple essential HSV-1 genes in comparison to single genes [120,122].

Our studies demonstrated the complete suppression of virus replication and reproduction with the use of a plasmid-encoded CRISPR/Cas9 system [113,114]. For example, HSV-1 replication and reproduction were inhibited in Vero cells transfected with a plasmid carrying CRISPR/Cas9 targeting *UL29* and *UL52* for a period of 48 h following infection. Additionally, our findings indicated that virus reproduction exhibited a gradual recovery, reaching a level that was only half that of control cells infected with a vector lacking the sgRNA after 144 h [114]. Furthermore, a pair of sgRNAs targeting *UL19* and *UL30* was identified, which resulted in a complete blockage of infection after 48 h post-infection. Nevertheless, complete inhibition of infection was also observed with an individual anti-HSV-1 sgRNA targeting *UL30* but not *UL19*. Notably, the longest complete inhibition of viral reproduction at 144 h after infection was achieved with an individual anti-UL30 sgRNA [113]. Earlier screening of HSV-1 gene editing targets by other authors also revealed that anti-UL30 sgRNAs had the most pronounced antiviral effect [120].

Thus, in vitro tests demonstrate that the CRISPR/Cas9 system has the potential to serve as an effective tool for inhibiting lytic infection caused by HSV-1. The more challenging task of blocking latent infection and reactivation of HSV-1 is of particular interest. To date, only a limited number of studies have assessed the capacity of the CRISPR/Cas9 system to impede the replication and reactivation of HSV-1 in latently infected cells. Utilization of the CRISPR/Cas9 system during lytic HSV-1 infection has been observed to induce more extensive rearrangements of the viral genome than during latency, with the presence of short indels near the editing site being a notable phenomenon [120]. Furthermore, the authors demonstrated that editing the *UL29* and *UL30* genes in an in vitro model of latent HSV-1 infection resulted in a reduction in virus reactivation, with a more pronounced effect observed with the use of dual anti-HSV-1 sgRNAs [120]. Similarly, van Diemen et al. found that targeting CRISPR/Cas9 against the *UL8*, *UL29,* and *UL52* genes in a model of latent HSV-1 infection successfully blocked reactivation. However, an analysis of virus replication levels and virus sequencing for anti-HSV-1 genome editing assays indicated that editing likely occurs only in newly synthesized viral genomes in sporadic, spontaneously reactivated cells and not in latent genomes [112].

### 3.2. The Second Direction: The Potential of the CRISPR/Cas9 System as an Anti-HSV-1 Therapy

#### 3.2.1. Standard Chemotherapy Against HSV-1

Before discussing the use of the CRISPR/Cas9 system as a therapy for infections caused by HSV-1, let us briefly review the currently used standard chemotherapy against HSV-1 (Table 2).

At present, numerous therapeutic drugs against herpesviruses are known, each with a distinct mechanism of action [129]. However, not all of these have been approved for clinical use. The majority of drugs employed in the treatment of HSV-1 are nucleoside analogs that exert their effect by interfering with the viral DNA polymerase encoded by the *UL30* gene, thereby inhibiting viral replication and preventing the formation of new viral particles. It is a prerequisite for all nucleoside analogs to undergo tri-phosphorylation prior to binding to and inhibiting the viral DNA polymerase. ACV and famciclovir facilitate the initial phosphorylation in collaboration with the viral TK, which is encoded by the *UL23* gene. Subsequently, cellular kinases become involved in the cascade of phosphorylations. In conclusion, the resulting triphosphate is incorporated into the formation of the viral DNA polymerase, which, in turn, by breaking the chain of viral DNA, inhibits its further replication. The advantage of this large group of drugs is that they have relatively low toxicity for patients [129]. However, the most significant challenge associated with this class of drugs is the emergence of mutant strains of the virus that exhibit resistance to nucleoside analogs. In the majority of cases, approximately 95% resistance or reduced sensitivity of the virus can be attributed to mutations in the viral TK. The most prevalent mutations that lead to resistance are AA substitutions in ATP and nucleoside binding sites, as well as insertions or deletions in G/C homopolymer sequences that result in frameshifts or the appearance of premature stop codons. In less common instances, these may manifest as substitutions of individual amino acids in regions of the ATP binding site or the nucleoside binding site that are considered to be conservative. It is also possible that mutations in the viral DNA polymerase gene may result in resistance, although this is an uncommon occurrence, accounting for approximately 5% of cases [130].

Foscarnet is a pyrophosphate analog that does not necessitate the involvement of TK, as it directly and reversibly binds to the viral DNA polymerase. Its mechanism of action is to bind to the pyrophosphate binding site, thereby preventing nucleotides from binding and incorporating them into the growing viral DNA chain. This is why it has been demonstrated to be an efficacious therapeutic agent for the treatment of ACV-resistant strains of HSV-1. However, its low selectivity, which results in binding to the active sites of all polymerases, creates the possibility of inhibiting human DNA polymerase. The high nephrotoxicity and other side effects associated with foscarnet limit its use in many patients. Additionally, resistance to foscarnet has been observed in mutant strains of HSV-1, which typically occurs at the pyrophosphate binding site of the viral DNA polymerase [129].

A novel class of antiherpetic drugs has been identified, comprising helicase (UL5) and primase (UL52) inhibitors. Amenavir and Pritelevir are currently undergoing clinical trials. The precise molecular mechanism by which they prevent the formation of the helicase–primase complex remains unclear. However, it is known that certain mutations in the HSV-1 population can lead to resistance of the virus to the action of these drugs [11,129].

An increasing number of reports are emerging that suggest potential effective solutions in the field of gene therapy for diseases, including viral ones. In recent studies, CRISPR/Cas9 has demonstrated efficacy as a treatment for HSV-1 in vitro and in vivo [111,123,131]. It is also noteworthy that the currently approved medications do not address the issue of latency-reactivation of HSV-1. Concurrently, gene therapy offers optimism for favorable developments in this regard [123]. The available data on the use of CRISPR/Cas technology to treat infections caused by HSV-1 are discussed in the next section.

#### 3.2.2. HSV-1 Targeting In Vivo by CRISPR/Cas9 System

The research into the efficacy of anti-HSV-1 therapies in vivo is progressing rapidly. Ying et al. demonstrated that intracerebral injection of lentiviral vectors (LVs) carrying components of the CRISPR/Cas9 system targeting HSV-1 genes five days prior to HSV-1 infection resulted in the inhibition of viral spread in the brains of mice. HSV-1 spread is most effectively blocked by using sgRNAs against *ICP4* and *ICP27* [111]. In another study, the influence of HSV-1 gene deletions obtained by the CRISPR/Cas9 system on the course of latent infection was investigated. The *UL7* knockout was observed to cause a two-fold decrease in LAT mRNA expression in the spinal cord and trigeminal nerve of mice in the model of latent HSV-1 infection in vivo [116].

In a study conducted by Yin D. et al., the CRISPR/Cas9 system was employed for the treatment of herpetic eye diseases in mice [122]. The authors constructed an sgRNA expression cassette targeting two essential HSV-1 genes, *UL8* and *UL29*, and co-packaged it with *Staphylococcus aureus* Cas9 (SaCas9) messenger RNA (mRNA) in lentiviral vectors (LVs). The resulting system was designated as HSV-1-erasing lentiviral particles (HELP). To assess the preventive action of HELP, lentiviral particles were administered by intrastromal injection to the corneas of mice one day prior to HSV-1 infection. To ascertain whether HELP treatment impedes the transmission of HSV-1 from the corneal epithelium to the peripheral and central nervous system (CNS), eyes, TGs, and brain, samples from all infected mice were harvested and examined for the copy number of the HSV-1 genome and infectious viruses. A significant reduction in viral load was observed in all samples following HELP treatment. All mice in the HELP-treated groups survived, and no relapse of HSV-1 was observed in the HELP-treated mice during the three-month follow-up period. Experiments have demonstrated that HELP is capable of curing HSV-1 in mice in both therapeutic and recurrent models. Therefore, the efficacy and safety of HELP, as demonstrated by Yin D. et al., indicates the potential for further clinical testing of the system.

In a recent publication, Wei A. et al. [131] described the first in vivo application of CRISPR/Cas9 to treat patients with herpetic stromal keratitis HSK (this paper is based on clinical trial NCT04560790). HSK is a severe ocular disease that is the leading cause of blindness. It is estimated that 1.5 million new cases of HSK occur annually [132]. ACV treatment has been associated with complications due to the drug’s high toxicity. Additionally, the radical treatment method of corneal transplantation has been found to be ineffective in addressing HSV-1 infection recurrence, with a high incidence (up to 55%) of recurrence reported in some cases [133]. Previously, CRISPR/Cas9-based HELPs [134] have been employed to effectively target vascular endothelial growth factor A in a mouse model of wet age-related macular degeneration. Wei A. et al. were the first to employ HELP in the treatment of HSK in patients [131]. The HSV-1 *UL8* and *UL29* genes, which are involved in virus replication, were selected as targets for CRISPR/Cas9 in vivo. To prevent the unintended integration of the lentivirus, the authors introduced a D64V mutation into the integrase gene. HELP was employed to treat three patients with HSK by injecting particles into the recipient graft bed at six to eight sites following corneal transplantation. Quantitative PCR analysis demonstrated a reduction and subsequent elimination of HSV-1 DNA in corneal samples from all patients, including one with a markedly elevated viral load in the cornea prior to treatment. It is noteworthy that HSV-1 DNA was undetected (Table 2) even 21 months following a single dose of HELP, indicating that viral activity had not recurred. A special analysis demonstrated the absence of off-target cleavage in the human genome and the absence of notable CRISPR-related side effects following HELP administration [131]. These results suggest that CRISPR/Cas9-based treatment may be an effective and safe approach to control infectious viral keratitis and may also have good potential for the treatment of other HSV-1-related diseases.

This clinical trial has now been completed, and a Phase 1 clinical trial (NCT06474416) has been initiated with the objective of determining the safety, tolerability, and preliminary efficacy of the treatment. The study is scheduled to conclude in 2025, at which point Phase IIa (NCT06474442) will be initiated with the objective of determining the safety, tolerability, and efficacy of CRISPR/Cas9 therapy. CRISPR/Cas9-related technologies are advancing towards clinical trials, including for the treatment of viral infections. In addition to clinical trials for HSV-1, CRISPR/Cas9 technology is being tested for other viruses, including human papillomavirus, human immunodeficiency virus 1 (HIV-1), Epstein–Barr virus, and the novel coronavirus SARS-CoV-2. It is, therefore, probable that CRISPR-Cas9 technology will be widely tested and used for the therapy of viral diseases in the near future.

#### 3.2.3. Improving the Delivery of CRISPR/Cas Systems In Vivo

Earlier in vivo studies and the initial in vivo therapeutic applications of CRISPR/Cas9 indicate that the efficacy of in vivo genomic editing is contingent upon the efficiency of CRISPR/Cas9 delivery. The absence of efficient, reliable, and safe in vivo delivery methods represents a significant obstacle to the clinical application of the CRISPR/Cas9 system [135,136]. The CRISPR/Cas9 delivery methods currently under development can be broadly categorized into three main groups: biological, chemical, and physical [137,138]. Biological methods employ the use of naturally occurring biological materials, such as viral proteins, peptides, or cellular receptors, to facilitate penetration of the cell. Viral vectors, virus-like particles (VLPs), and cell-penetrating peptides are examples of this category. Chemical methods employ artificially synthesized materials, including polymers, lipids, and metals. These include liposomes, gold nanoparticles, and lipid nanoparticles. Physical methods rely on the use of electrical or ultrasound energy for delivery into cells. Examples of this category include electroporation, sonoporation, and microinjections. Each delivery system possesses both advantageous and disadvantageous characteristics, necessitating further refinement for therapeutic applications.

At present, viral vectors are regarded as the most efficacious method of delivering CRISPR-Cas components into host cells, given that viruses have evolved mechanisms to facilitate efficient infection of a wide range of human tissues and cells. Moreover, the virion structure safeguards the target molecules (cargo) from degradation by host enzymes within cells and throughout the body. Viruses, including recombinant viruses, gain access to target cells and replicate, thereby ensuring the sustained expression of the delivered molecules. It is crucial to emphasize that, for reasons of safety, any pathogenic or virulent genes must be removed from the vector genome. The replication of the viral vector can be prevented by the deletion of the genes involved in this process, thus preventing the replication of the vector in the target cells [138]. Viral vectors that have been extensively studied, including adeno-associated viral vectors (AAV), lentiviral vectors, and full-length adenoviral vectors, are widely used for in vivo delivery due to their ability to incorporate therapeutic genes into their genome or encapsulate genetic materials to facilitate intracellular delivery [139]. AAV vectors require a more detailed description as they are currently FDA approved and used in numerous clinical trials [140,141].

AAVs are small, nonpathogenic viruses belonging to the family *Parvoviridae*, which includes viruses with single-stranded, uncoated DNA. Notwithstanding the high prevalence of these viruses in the human population (approximately 80% of individuals are seropositive for AAVs), they are not associated with any human disease. AAVs are currently the leading in vivo delivery system for CRISPR components compared with other viral delivery methods due to their low immunogenicity and cytotoxicity, as well as their limited integration into the host cell [135,142]. To prevent the integration of AAV into the host genome, recombinant AAV vectors (rAAV) have been developed in which the replication (Rep) genes encoding the proteins necessary for viral DNA replication, modulation of viral gene expression, and site-specific integration into the host genome have been removed [143]. Another significant advantage of AAV vectors over other viral vectors is the existence of multiple known AAV serotypes that are specific to different tissues. These include lung epithelial cells, heart cells, neurons, and skeletal muscle cells, which greatly facilitate tissue-specific transgene delivery [144]. In recent years, there has been a notable focus on the application of gene editing technologies for the purpose of inactivating and eliminating viral reservoirs that contribute to persistent or chronic infections [141,145,146]. The direct delivery of viral-specific gene editing enzymes or RNA interference molecules for the purpose of destroying or inactivating viral reservoirs represents a potentially therapeutic strategy for persistent viral infections that affect billions of people worldwide. AAV represents a promising delivery vector for the antiviral therapy of persistent and chronic infections. AAV vectors with high tropism and high transduction efficacy in the peripheral nervous system, in the liver, and in CD4+ T cells, in the latency sites of HSV-1 and HSV-2, varicella-zoster virus, hepatitis B virus, and HIV-1 have been obtained [141,145,147].

Despite the remarkable success of AAV vectors, there remain obstacles to their use, mainly related to the large molecular size of traditional CRISPR/Cas nucleases, which exceeds the packaging capacity of AAV vectors [148]. Researchers have devised several strategies to address this challenge. One approach involves using AAVs to deliver only sgRNA into cells that have previously been transduced with Cas9. Another method entails encapsulating sgRNA and Cas9 in separate AAVs with distinct tags, co-transducing them into cells, and subsequently screening the cells to assess the outcomes of co-transduction [149]. However, the delivery of two AAVs necessitates a high dose of virus, which gives rise to safety concerns [150]. An alternative approach is to utilize smaller orthologs of Cas9 and an alternative class of effector proteins in lieu of SpCas9. Smaller Cas9 orthologs, such as those derived from *S. aureus* (SaCas9) and *Streptococcus thermophilus* (St1Cas9), facilitate the packaging of sgRNA and Cas9 into a single AAV [148,151]. Recently, an alternative effector protein, CasX (Cas12e), isolated from *Deltaproteobacteria* (DpbCasX), has been the subject of study [152]. It is one of the smallest and most accurate genome editors to date [153]. For the first time, we have demonstrated that CRISPR/CasX systems targeting critical regions of the HSV-1 *UL30* gene can provide effective and long-term suppression of HSV-1 infection in vitro [113]. These findings suggest that CasX is a promising genome editor that could be employed to develop novel therapeutic strategies to combat HSV-1 infection and potentially other viral infections.

One of the most challenging aspects of clinical testing of AAV vectors delivering CRISPR/Cas9 is the presence of pre-existing antibodies against Cas9 and the subsequent immune response to sgRNAs [154]. It should be noted that all viral vectors, not merely AAV vectors, have the potential to induce Cas9-specific immunity [155,156]. The reduction of immune recognition can be achieved through the engineering of Cas proteins, the utilization of CRISPR/Cas systems derived from non-pathogenic organisms, the induction of immune tolerance, or the shielding of Cas proteins in the systemic circulation [157]. The AAV capsid protein, which constitutes the outer envelope of the virions, is the primary determinant of immunogenicity. The production of neutralizing antibodies against the AAV capsid can have a number of deleterious consequences, including the obstruction of delivery, the induction of acute inflammation, or the destruction of cells subsequent to editing [142,158]. At present, the immunogenic AAV capsid protein can be modified by altering the antigenic sites in order to reduce the affinity of binding to antibodies and thus circumvent the host immune response. Furthermore, the successful delivery of CRISPR/Cas9 in vivo may be facilitated by the plasticity and diversity of AAV capsid serotypes [159]. Consequently, while some successes in in vivo gene editing have been achieved using viral CRISPR/Cas delivery methods, the existing safety problems have prompted the search for alternative, non-viral delivery systems for the CRISPR/Cas9 system into body cells.

To address the limitations of viral vectors, a viral-like particle (VLP) system was developed that utilizes a minimal number of viral components. Viral capsids are produced by the self-assembly of viral structural proteins [160]. In terms of their external appearance, VLPs are similar to native viruses. However, they lack the viral genome, which means that they are unable to replicate [161]. The absence of replication in the host cell permits the classification of the VLP system as a form of non-viral delivery. Despite their relatively short life cycle, VLPs have attracted considerable attention with regard to vaccine development [162]. Transient delivery systems, including VLPs, provide genome editing while circumventing the potential complications associated with prolonged Cas9 expression. Consequently, VLPs and other non-viral carriers are emerging as promising candidates for therapeutic applications, particularly in comparison to other conventional viral vectors. Clinical trials (NCT06474416, NCT06474442) using VLP to treat HSK by Shanghai BDgene are currently underway [139].

The Cas9 protein and sgRNA are susceptible to rapid degradation following systemic injection, necessitating the protection of these components for in vivo applications. In a seminal study, Mount et al. demonstrated a highly efficient editing strategy based on co-targeted delivery of Cas9 protein and sgRNA into cells. Gold nanoparticles were employed for the coassembly of Cas9 and sgRNA in nanoassemblies. The vectors demonstrated the capacity to efficiently deliver the protein and nucleic acid into the cytoplasm, subsequently facilitating transport to the nucleus. The authors achieved delivery efficiency of approximately 90% into various cell types, with gene editing efficiencies of up to 30% [163]. It is important to note, however, that this method does not fully protect Cas9 protein and also suffers from other drawbacks, such as immunogenicity, toxicity, and rapid clearance when administered systemically. 

In recent years, extracellular vesicles (EVs) have emerged as a promising delivery system for a diverse range of molecules and microstructures, including proteins, RNA, DNA, and even virions. They are markedly more secure and exhibit superior pharmacokinetics when compared to synthetic nanocarriers [164]. Extracellular vesicles (EVs) are naturally occurring nanoparticles secreted by a multitude of cell types. They exhibit high biocompatibility and are capable of traversing biological barriers [165]. The use of CRISPR/Cas systems packaged in EVs has proven effective in the creation and treatment of animal models of human diseases [165]. Extracellular vesicles (EVs) produced by cancer cell lines display a natural tropism for their respective tumors [166]. In general, the tropism of EVs can be modified through genetic engineering of receptor proteins or modification of already assembled particles. The presented data suggest that EVs are a versatile delivery system for CRISPR/Cas systems with potential for therapeutic applications.

Further improvements to the delivery methods for the CRISPR/Cas9 system, as previously discussed, should enhance the efficacy of therapies for HSV-1 infections in vivo.

#### 3.2.4. Disadvantages of CRISPR/Cas Systems as Anti-HSV-1 Therapeutics

Despite significant advances in the adaptation of CRISPR/Cas systems in human disease therapy [167], including the FDA-approved CRISPR/Cas9-derived cell product Casgevy for the treatment of sickle cell anemia [168], there are inherent shortcomings of CRISPR/Cas systems as therapeutic tools that must be overcome in order to create effective and safe therapies for both genetic and viral human diseases. 

First of all, CRISPR/Cas systems, especially CRISPR/Cas9, are known for their off-target activity, i.e., their ability to induce sequence changes in other regions of the genome that are not fully complementary to the spacer sequence of the guide RNA [169]. In extreme cases, the number of mismatches between the spacer and off-target can be six [170]. Off-target activity may be one of the reasons why CRISPR/Cas systems are toxic to cells [171]. Previously, we observed decreased survival of Vero cells transfected with plasmids, with the CRISPR/Cas9 system encoding some spacers against HSV-1 genes *UL19* and *UL35* or a combination of two spacers against *UL19*/*UL30* genes [113], whereas the survival of cells with spacers against other HSV-1 genes was unchanged compared to negative controls. These data indicate that some spacers against targets in the HSV-1 genome may be toxic to mammalian cells, requiring preliminary tests to select safe spacers. Possible solutions to this drawback include the use of more precise mutant variants of Cas effectors [172], as well as the use of approaches that allow short-term expression of components of CRISPR/Cas systems in mammalian cells.

In addition to off-target effects, there are adverse on-target effects of CRISPR/Cas systems that could result in the loss of a large chromosome fragment (loss of heterozygosity) as well as large-scale chromosomal rearrangements (chromothripsis) [173,174]. In the case of a viral genome, this would be advantageous in that it could result in the loss of essential viral genes and the elimination of the virus from the cell in the majority of cases. Nevertheless, it is conceivable that CRISPR/Cas-induced recombination of the damaged viral genome with a non-targeted copy of the viral genome or constrained action of mutagenic DNA repair systems could result in the repair of indels or potentially the loss of the CRISPR/Cas system target while the targeted viral gene undergoes tolerable mutations. This represents a potential mechanism for the emergence of escape mutant viral strains, as observed in the case of HIV-1 [175]. It is, therefore, possible that the application of the CRISPR/Cas9 system may also result in the generation of escape mutants of HSV-1. One potential solution to this problem is the use of a combination of sgRNA spacers [176]. In the case of HSV-1, this solution is also effective, as evidenced by the increased efficiency of CRISPR/Cas-mediated suppression of HSV-1 infection observed when a combination of two, three, or four spacers targeting different viral genes is used [110,177]. Nevertheless, we have shown that there is a site in critically important HSV-1 genes, such as *UL30*, whose targeting by even a single spacer provides long-standing suppression of HSV-1 infection [113]. Nevertheless, we have shown that there is a site in critically important HSV-1 genes, such as UL30, whose targeting by even a single spacer provides long-lasting suppression of HSV-1 infection. Note that the use of a single spacer is safer than two or more when using the CRISRP/Cas9 system.

Another issue with using CRISPR/Cas systems as a therapeutic tool is their high immunogenicity since Cas effectors are rather large proteins of bacterial origin [178]. Therefore, genetic constructs (plasmids, viral vectors) lead to prolonged expression of Cas effectors, which is fraught with recognition of cured cells by the immune system and their subsequent destruction. Potential solutions include transient expression of Cas-effector, utilization of CRISPR/Cas as a ribonucleoprotein complex, mutation of immunodominant epitopes of Cas-effector, and targeting of immunoprivileged organs (e.g., eyes) [178].

We have discussed three key drawbacks of using CRISPR/Cas systems in the therapy of viral diseases and possible ways to overcome them. We believe that as CRISPR/Cas technology is further explored and improved in the future, it can reach a level of relative safety for use in the therapy of human hereditary and viral diseases.

### 3.3. The Third Direction: CRISPR/Cas-Based HSV-1 Detection

A clinical diagnosis of herpesvirus infections does not always permit the differentiation of infections caused by HSV-1 from infections exhibiting similar clinical manifestations but caused by other pathogens of disparate natures. Consequently, rapid diagnosis is essential for the timely prescription of specific antiviral treatment. The monitoring of the causative agent can inform management decisions in high-risk patients and may contribute to reducing hospital stays, thereby reducing treatment costs and the burden on the healthcare system [179]. At present, a range of techniques are available for the laboratory diagnosis of HSV-1 infection. These methods can be classified into several principal categories:

1. Serologic: anti-HSV-1 IgM and anti-HSV-1 IgG assays.
2. Immunocyto- and histochemical techniques are employed for the detection of viral antigens (proteins).
3. Culture: the detection of infectious active virus and the isolation of HSV-1 from clinical samples.
4. Molecular–biological: the detection of viral DNA and individual nucleotide sequences.

Each diagnostic group includes methods used to diagnose HSV-1 infection, which are summarized in Table 3 and are discussed below.

#### 3.3.1. The Serological Diagnosis of Herpesvirus Infections 

These methods include the use of enzyme-linked immunosorbent assay (ELISA) to detect anti-HSV-1 and anti-HSV-2 immunoglobulin M (IgM) and immunoglobulin G (IgG) antibodies in peripheral blood. Given the high degree of DNA genome homology between the two herpesviruses, some existing tests concentrate on the detection of antibodies to shared determinants of HSV-1 and HSV-2 (HSV1/2), while others differentiate antibodies to each virus. The choice of specific tests to be employed is dependent on the localization and manifestation of the clinical signs. The two types of tests permit the determination of the stage of infection at the time of examination and the monitoring of the infectious process. The presence of IgM antibodies to HSV-1/2 indicates a primary infection or reactivation of the latent virus in the early stages of relapse. Conversely, the presence of IgG antibodies to HSV-1/2 indicates a current infection. Furthermore, IgG antibodies to HSV-1/2 can be detected in patients with a history of disease and may persist for many years or indefinitely. This makes it challenging to differentiate between current and past infections without additional methods. One such method is the determination of antibody avidity, which is employed in cases where the results of anti-HSV1/2 IgG or IgM tests are inconclusive. This approach is used to exclude or confirm the likelihood of a recent primary infection, as antibodies with low avidity (with an avidity index of 30–40%) indicate a recent infection. Another method for confirming serological results is immunoblotting (Western blot, WB), which detects antibodies to multiple viral proteins in the patient’s blood. This assay is highly sensitive and specific, but it is labor-intensive and expensive, and it is used in cases where the results of ELISA are uncertain. For example, M.R. Golden et al. demonstrated that 11 of 70 individuals with indeterminate HSV-2 ELISAs were Western blot-positive [183]. In a recent study, Crawford KHD et al. (2024) conducted a comparative analysis of three automated serologic tests for anti-HSV-1 and anti-HSV-2 IgG, which have been approved by the Food and Drug Administration (FDA), in conjunction with Western blotting across nearly 2000 samples. It is noteworthy that the study samples included those with PCR or culture-confirmed HSV-1 infection. The authors concluded that routine methods for serological diagnosis of HSV-1 have limitations (low sensitivity and specificity) that prevent their widespread use and recommended an algorithm for confirming serologic diagnoses of HSV-1 infections [182]. Nevertheless, serological diagnosis remains a valuable tool for both clinicians and screening studies. Therefore, further improvements in the serological diagnosis of herpesvirus infections of HSV-1 etiology are a relevant area of research.

#### 3.3.2. HSV-1 Antigen Detection in Clinical Specimens

The developed methods, namely immunofluorescence assays (IFA) and immunoperoxidase analysis (EIA) in situ using monoclonal antibodies (mAbs), permit the detection of HSV-1 antigens in the tissues and cells of patients. This approach offers several advantages, including low cost and rapid results (within two hours), as well as the ability to identify individual virus-infected cells in clinical samples. Furthermore, when used in conjunction with high-resolution microscopy, the method enables the localization of viral proteins within intracellular structures. However, the dependence of the results on the quality of the material collected and the qualifications of the researcher restrict the method’s applicability in broad virological practice [185,186].

#### 3.3.3. Detection of HSV-1 by Culture Methods

Virus isolation in cell culture has long been regarded as the gold standard for diagnosis, as it provides definitive proof of the presence of an infectious pathogen in the patient. This is evidenced by numerous studies [195,196]. HSV-1 can be isolated from a variety of biological fluids. The fulfillment of certain conditions, the availability of appropriate materials, and the utilization of cell culture equipment are prerequisites for this process. It is imperative that clinical samples containing live viruses be transported to the laboratory for a brief period, with the utmost care taken to maintain a cold chain at 4 °C. Following the introduction of samples into sensitive cells in culture, the specific cytopathic effect (CPE) of HSV-1 typically manifests within three to four days. However, in some instances, this may extend to seven or more days, contingent on the quantity of virus present and its activity within the sample. Virus isolation is, therefore, a time-consuming and labor-intensive process, but it has the major advantage of demonstrating the presence of infectious virus within a clinical lesion and also allows for sensitivity testing to antiviral drugs.

To address one of the limitations of the culture method, namely the length of the assay, a modified rapid culture method (RCM) was developed. The procedure entails fixing the cells 24 to 48 h after the introduction of clinical samples, prior to the emergence of CPE, followed by treatment with monoclonal antibodies (mAbs) specific to HSV-1. The determination of specific antigen (Ag) in cell culture by RCM is not only a more rapid method but also one that is more sensitive for the detection of infectious viruses in biological material than the classical culture method [189]. The available data confirm that RCM has both a high diagnostic specificity and a high prognostic value of 100% and 94%, respectively.

#### 3.3.4. Molecular Biological Methods—Detection of Virus Nucleic Acids by PCR

At present, the polymerase chain reaction (PCR) is a widely utilized method of nucleic acid amplification in practical medicine for the detection of HSV1/2. A variant of the PCR method that has gained considerable popularity is real-time PCR (quantitative version). This variant is employed for diagnostic purposes [190] and for determining sensitivity to antiviral drugs. The classical variant of the PCR method is currently employed extensively in clinical virology for the screening and monitoring of HSV-1 infections [187]. The method offers high sensitivity, specificity, and rapidity of study. The PCR method is currently regarded as the gold standard for the detection of HSV-1 DNA in CSF and CNS [197,198]. The sensitivity of the method is 98% to 100% [188]. It should be noted that the aforementioned indicator may vary depending on the specific type of tested samples and the potential presence of inhibitors. One of the method’s shortcomings is its lack of standardization. The generally accepted quantitative parameters corresponding to clinically significant manifestations of infection, as well as values characterizing different stages of the disease, have yet to be determined. The high sensitivity of the method presents both advantages and disadvantages. PCR detects the DNA of a latent virus, which limits the predictive value of the method. Different researchers have found that only 40% to 60% of patients with detected HSV-1 DNA by PCR develop an active form of HSV-1 infection. Conversely, a negative PCR result has a 100% diagnostic value [188].

#### 3.3.5. Molecular Biological Methods—CRISPR/Cas-Based Diagnosis of Viral Infections

CRISPR-based diagnostic assays have emerged as a promising avenue for infectious disease diagnosis due to a number of factors, including their simple design, rapid implementation, low cost, and high scalability. The most commonly used CRISPR system, Cas9 protein, has been largely overlooked in the field of diagnostics due to its propensity for off-target activity and the inherent challenges associated with conducting and interpreting the results of such experiments [199]. The selection of the optimal CRISPR-associated Cas protein from the numerous currently known and described in this review is a crucial aspect of the success of CRISPR diagnostics. Advances in CRISPR diagnostics are linked to the development of digital CRISPR systems for the quantitative detection of nucleic acids. A variety of methods, including SHERLOCK, HUDSON, HOLMES, and DETECTR, have been developed by coupling isothermal amplification and CRISPR/Cas sensing to detect a range of viruses [193,200]. Wu X. et al. have developed the warm-start rapid digital CRISPR approach (WS-RADICA), which enables rapid, sensitive, and quantitative detection of nucleic acids [193]. The WS-RADICA method is suitable for the quantitative determination of DNA and RNA with high sensitivity, as evidenced by the ability to detect as little as one copy per milliliter of SARS-CoV-2 RNA. The WS-RADICA method incorporates digital PCR (dPCR), which exhibits greater tolerance to inhibition than qPCR. A comparison of the results obtained with the QIAcuity Digital System demonstrated that the WS-RADICA can be readily adapted to a range of digital devices. The authors evaluated the system’s capabilities for analyzing two DNA genomic viruses: adenovirus and HSV-1. The combination of a loop-mediated isothermal amplification (LAMP) primer set and a crRNA with the highest speed was selected for the subsequent WS-RADICA experiment. The DNA extracted from human adenovirus (hAdV) and HSV-1 was serially diluted (hAdV: 2.6 to 5612 copies/mL; HSV-1: 1.6 to 3506 copies/mL) and tested by the respective WS-RADICA assay. The ratio of positive partitions increased in a linear fashion with the increase of target DNA for both viruses under study. The high sensitivity and rapid detection (within one hour) of hAdV and HSV-1 DNA indicate the ability of WS-RADICA to quantify real viruses in culture cells and clinical samples. The specific high-sensitivity enzymatic reporter unlocking (SHERLOCK) and PCR-LwCas13a methods have demonstrated remarkable efficacy in pathogen detection by integrating recombinase polymerase amplification (RPA) or PCR with Cas13a technology [201]. The SHERLOCK method has been employed for the detection of outbreaks of RNA viruses, including Lassa fever, Ebola, Zika, and dengue. Additionally, it has been successfully validated for the detection of SARS-CoV-2, lymphocytic choriomeningitis virus, influenza A virus, HIV-1, and vesicular stomatitis virus [202,203,204]. In a recent publication, Dou et al. proposed a novel photoelectrochemical method for the detection of HSV-1 using multiple potential step chronoamperometry (MUSCA), which is based on the CRISPR/Cas12a cleavage coupled with entropic target recycling. The experimental data demonstrate the specificity, long-term stability, and reproducibility of the CRISPR/Cas12a-based MUSCA-PEC strategy. This strategy has been successfully applied to detect HSV-1 DNA in human serum with high sensitivity (1 pM). The combination of the MUSCA method and the CRISPR/Cas12a assay holds greater promise for HSV-1 detection [191]. In 2024, a CRISPR-Cas13a-based isothermal assay for the detection and genotyping of HSV-1 was developed [194]. The method allows for the detection of a single copy of viral DNA per microliter and the interpretation of results via a mobile application or calorimetric analysis. The assay demonstrated a high sensitivity of 96.15% and 95.15% for HSV-1 and HSV-2, respectively, as well as 100% specificity in comparison to a commercial quantitative PCR assay. Therefore, although the CRISPR system is not yet approved by the FDA for diagnostic purposes, it may serve as a promising tool for monitoring and detecting infectious human viral diseases.

Despite the extremely high sensitivity of some diagnostic test systems developed with CRISPR/Cas, research continues, as many issues of diagnosis of viral infections, including HSV-1 infections, have not yet been fully resolved.

### 3.4. The Fourth Direction: CRISPR/Cas-Based Validation of Drug-Resistant HSV-1 Mutations

In individuals with compromised immune systems, long-term anti-HSV-1 treatment is frequently necessary, which may contribute to the emergence of drug-resistant viruses. ACV resistance is primarily attributed to TK mutations, though DP mutations have also been observed on occasion [205]. The CRISPR/Cas9 system offers a valuable approach for investigating mutations in the relevant genes.

The evolution of HSV-1 was observed in an immunocompromised patient [206]. The genome of HSV-1 isolates from the patient was sequenced, revealing the presence of ACV-resistant mutations in the TK gene (*UL23*). Furthermore, a novel Q727R mutation in the DNA polymerase gene (*UL30*) was identified, which confers multidrug resistance (ACV/foscarnet/adefovir). The role of this mutation in drug resistance was validated by introducing the Q727R mutation to the wild-type HSV-1 genome through cotransfection of plasmid DNA with CRISPR/Cas9 and an asymmetric ssDNA for homologous recombination template in HSV-infected cells, with the addition of the NHEJ inhibitor SCR7. This resulted in the generation of a de novo mutant that acquired multidrug resistance.

In a recent study, an ACV-resistant strain of HSV-1 was isolated from a patient with herpetic eye disease [207]. The authors identified a novel F289S mutation in the *UL23* gene. To substantiate the contribution of this mutation to the ACV resistance exhibited by the clinical isolate, the mutation was reversed to the wild-type through the use of CRISPR/Cas9-mediated homology repair. This was accomplished by employing CRISPR/Cas9 ribonucleoprotein complexes that target UL23 in proximity to the F289S substitution site in conjunction with a double-stranded DNA repair template. This resulted in the restoration of sensitivity to ACV. In contrast, the introduction of the F289S mutation into the wild-type strain by CRISPR/Cas9-mediated homology repair resulted in the development of ACV resistance.

The two articles illustrate the effectiveness of the CRISPR/Cas9 gene editing tool in confirming the role of detectable mutations in HSV-1 clinical isolates in the emergence of drug resistance.

## 4. Conclusions

CRISPR/Cas systems have demonstrated considerable potential in a range of applications, including the detection of HSV-1, the validation of mutations in the HSV-1 genome that lead to drug resistance, and the development of therapeutic agents for the treatment of HSV-1 infections. Nevertheless, several obstacles must be surmounted before an effective and secure therapeutic-grade CRISPR/Cas system can be developed. It is anticipated that further development and improvement of components and delivery methods will enhance the efficacy and safety of the CRISPR-Cas system for the treatment of human viral diseases, including those caused by HSV-1.

## Figures and Tables

**Table 1:** HSV-1 genes used as targets of CRISPR/Cas9 system.

Targeted Gene (Protein Name)	Function of Gene	Name of Cas Nuclease	Method or Vector Delivery	Anti-HSV-1 Activity	Reference
* Immediate early genes					
*RL2* (ICP0)	RING-type E3 ubiquitin ligase acts in initial stages of infection or during HSV-1 reactivation, helps to evade cellular antiviral response	SpCas9	Plasmid transfection	*ICP0*-mutants with indels demonstrated decreased reproduction	[110]
			LVs with sgRNA and cells expressing Cas9	Suppression of viral particle production	
			LVs with sgRNA and LVs with Cas9	Inhibition of viral replication and increased protection of cells from infection	
		SaCas9	AAV2 with sgRNA and SaCas9	Inhibition of viral replication in the Vero cell line and in cerebral organoids; suppression of the latent form of HSV-1 in cerebral organoids	[123]
		SaCas9	AAV9 with sgRNA and SaCas9	Inhibition of HSV-1 infection and reactivation in a latent rabbit keratitis model	[121]
		SaCas9 or SpCas9	LVs with SaCas9 or SpCas9 and sgRNAs	Protection of cells from infection	[119]
*RS1* (ICP4)	Major viral transcription factor	SaCas9	LVs with SaCas9 and sgRNAs	Inhibition of viral particle production	[120]
		SaCas9 or SpCas9	LVs with SaCas9 or SpCas9 and sgRNAs	Protection of cells from infection, reduction of viral infection	[119]
			AAVs with SaCas9 and sgRNAs	Inhibition of viral replication and particle production	
		SpCas9	Plasmid transfection	Inhibition of viral replication and particle production	[111]
*UL54* (ICP27)	Multifunctional regulator of viral and cellular gene expression, modulates splicing, 3′ processing, mRNA export, and inhibits host mRNA biogenesis	SpCas9	Plasmid transfection	Inhibition of viral replication and particle production	[111]
		SaCas9	LVs with SaCas9 and sgRNAs	Inhibition of viral particle production	[120]
		SpCas9	LVs with sgRNA and LVs with Cas9	Inhibition of viral replication	[112]
		SaCas9	AAV2 with sgRNA and SaCas9	Inhibition of viral replication in the Vero cell line and in cerebral organoids. Suppression of the latent form of HSV-1 in cerebral organoids.	[123]
		SaCas9	AAV9 with sgRNA and SaCas9	Inhibition of HSV-1 infection and reactivation in a latent rabbit keratitis model	[121]
*US12* (ICP47)	Multifunctional protein, blocks mRNA splicing, inhibits peptide presentation on MHC-I, transports viral mRNA from nucleus to cytoplasm	SpCas9	Plasmid transfection	The combination of *ICP47* and *ICP34.5* gene deletions suppresses viral replication and particle formation	[124]
Early genes					
*UL5*	Helicase, essential for HSV-1 genome replication	SpCas9	LVs with sgRNA and LVs with Cas9	Inhibition of viral replication	[112]
*UL8*	DNA helicase/primase complex-associated protein, essential for HSV-1 genome replication	SpCas9	LVs with sgRNA and LVs with Cas9	Inhibition of viral replication and particle production	[112]
		SpCas9	mRNA (Cas9)-carrying LVs with sgRNA	Inhibition of viral particle production	[122]
		SpCas9	Plasmid transfection	Targeting together with *UL29* reduces viral replication and particle production	[114]
*UL9*	Origin binding protein	SpCas9	LVs with sgRNA and LVs with Cas9	Inhibition of viral replication	[112]
*UL23*	TK	SpCas9		Inhibition of viral replication	[125]
*UL29* (ICP8)	ssDNA-binding protein	SpCas9	LVs with sgRNA and LVs with Cas9	Inhibition of viral replication and particle production	[112]
		SpCas9	mRNA (Cas9)-carrying LVs with sgRNA	Inhibition of viral particle production	[122]
		SpCas9	Plasmid transfection	Targeting together with *UL8* reduces viral replication and reproduction	[114]
*UL30*	DNA-polymerase	SpCas9	LVs with sgRNA and LVs with Cas9	Inhibition of viral replication	[112]
		SpCas9	Plasmid transfection	Reduction of viral replication and particle production	[113]
		DpbCasX	Plasmid transfection	Reduction of viral replication and particle production	
		SaCas9	LVs with SaCas9 and sgRNAs	Inhibition of viral particle production	[120]
*UL39* (ICP6)	Large subunit of ribonucleotide reductase; autophosphorylates via unique N terminus but does not trans-phosphorylate	SpCas9	Plasmid transfection	*UL39* knockout decreases HSV-1 replication, reproduction, and spreading	[115]
		SpCas9	Plasmid transfection	Inhibition of viral replication	[126]
*UL42*	Processivity factor, DNA-polymerase complex-associated protein, increasing the affinity of *UL30* for viral DNA	SpCas9	LVs with sgRNA and LVs with Cas9	Inhibition of viral replication	[112]
*UL48* (VP16)	Tegument protein, activates transcription of IE genes	SpCas9	Plasmid transfection	Inhibition of viral replication and particle production	[111]
*UL52*	DNA primase, essential for HSV-1 genome replication	SpCas9	LVs with sgRNA and LVs with Cas9	Inhibition of viral replication and particle production	[112]
		SpCas9	Plasmid transfection	Targeting together with *UL29* reduces viral replication and reproduction	[114]
*US3*	Viral serine/threonine kinase	SpCas9	LVs with sgRNA and LVs with Cas9	Inhibition of viral replication	[112]
Late genes					
*UL7*	Tegument protein involved in herpesvirus assembly	SpCas9	Plasmid transfection	*UL7* mutation (30 bp deletion) reduces replication and in vitro proliferation and decreases LAT mRNA levels in latent infection in mice	[116]
		SpCas9	Plasmid transfection	*UL7* mutation (30 bp deletion) attenuates pathogenicity, decreases replication, causes non-lethal infections in mice, lowers viral loads in brain and trigeminal nerve	[127]
*UL15*	Terminase complex protein	SpCas9	LVs with sgRNA and LVs with Cas9	Inhibition of viral replication	[112]
*UL16*	Tegument protein is crucial for the egress of capsids from the nuclei and the acquisition of a viral envelope	SpCas9	Plasmid transfection	*UL16* knockout decreases HSV-1 replication, reproduction, and spreading	[118]
*UL21*	Tegument protein, involved in cell-to-cell spread	SpCas9	Plasmid transfection	*UL21* knockout decreases HSV-1 replication, reproduction, spreading	[128]
*UL19* (VP5)	Major capsid protein, forms an icosahedral capsid with a T = 16 symmetry consisting of 162 capsomers	SpCas9	Plasmid transfection	Inhibition of viral replication and particle production	[113]
*RL1* (ICP34.5)	Neurovirulence factor	SpCas9	Plasmid transfection	*ICP34.5* knockout (replacement of the *ICP34.5* gene with a GFP expression cassette) inhibits viral replication and viral particle production	[124]
*UL35* (VP26)	Small capsomere-interacting protein, participates in the assembly of the infectious particles, forms a layer between the capsid and the tegument	SpCas9	Plasmid transfection	Effect is unknown due to high cellular toxicity of the CRISPR/Cas9 system targeted to the *UL35*	[113]
*UL36*	Inner tegument protein, deubiquitinase	SpCas9	LVs with sgRNA and LVs with Cas9	Inhibition of viral replication	[112]
*UL37*	Inner tegument protein involved in capsid traffic and virion morphogenesis	SpCas9	LVs with sgRNA and LVs with Cas9	Inhibition of viral replication	[112]
*UL41* (VHS)	Causes nonspecific degradation of mRNA after infection; shuts off host protein synthesis, enables sequential synthesis of viral proteins	SpCas9	Plasmid transfection	Attenuated pathogenicity (30 bp deletion in *UL7* gene and 59 bp deletion in the *UL41* gene), inhibition of viral replication, non-lethal infections in mice, lower viral loads in nervous tissues	[127]
*UL27* (gB)	Envelope glycoprotein that forms spikes at the surface of virion envelope, essential for the initial attachment to the host cell receptors, involved in fusion of viral and cellular membranes, together with gK, induces syncytia formation	SpCas9	LVs with sgRNA and LVs with Cas9	Inhibition of viral replication	[112]
*US6* (gD)	Envelope glycoprotein that binds to several cell receptors, including HVEM, NECTIN1, and 3-O-sulfated heparan sulfate	SpCas9	Plasmid transfection	Inhibition of viral replication and particle production	[111]
*US8* (gE)	Envelope glycoprotein is important for viral egress and cell-to-cell spread	SpCas9	LVs with sgRNA and LVs with Cas9	Inhibition of viral replication	[112]
		SpCas9	Plasmid transfection	*US6* knockout decreases HSV-1 replication, reproduction, and spreading	[117]
LAT	LAT transcript is responsible for HSV-1 latency establishment and maintenance	SpCas9	Plasmid transfection	Attenuated pathogenicity (30 bp deletion in *UL7* gene, 59 bp deletion in the *UL41* gene, and 138 bp deletion in the LAT gene), reduced replication, non-lethal infections in mice, reduced viral load in neural tissues and decreased latency	[127]

* Genes are labeled and grouped in the order of their temporal expression during the HSV-1 infection cycle, as described in Section 2.

**Table 2:** Comparison of the properties of standard therapeutic agents and the CRISPR/Cas9 system for the treatment of HSV-1 infections.

Name	Mechanism of Action	Advantages	Disadvantages	FDA Approval or Clinical Trials
ACV	Inhibits DNA polymerase indirectly through viral TK	Standard therapy, proven effectiveness	Neurotoxicity, virus can develop resistance	FDA approved
Foscarnet	Directly inhibits DNA polymerase by blocking the pyrophosphate binding site and preventing the cleavage of pyrophosphate from deoxynucleotide triphosphates	Useful for treating infections caused by ACV-resistant herpesviruses	Nephrotoxicity, wide range of side effects, virus can develop resistance	FDA approved
Amenavir, Pritelivir	Inhibition of the HSV-1 helicase–primase complex	Useful for treating infections caused by ACV-resistant herpesviruses	Virus can develop resistance	Phase 3 clinical trials (NCT03073967, NCT01959295)
CRISPR/Cas systems	Damage to viral DNA by introduction of double-stranded breaks followed by action by error-prone cellular DNA repair pathways, leading to inactivation of important viral genes	A potentially effective and safe approach to treat both active and latent HSV-1 infection	Insufficient knowledge of side effects	Phase 1, 2 clinical trials (NCT04560790, NCT06474416, NCT06474442)

**Table 3:** Laboratory methods for HSV-1 infection diagnosis.

Methods	Principle	Sample	Sensitivity/ Specificity	Advantages	Disadvantages	Reference
Serological methods Detection of antiviral antibodies	Anti-HSV-1 IgM ELISA	Serum, plasma,	88–95.4%/ 99%	Detection of early, acute phase of infection	False negative and false positive results; low sensitivity	[180]
	Anti-HSV-1 IgG ELISA		96%/ 86%	Markers of current, chronic, or latent infection	High prevalence in human population	[181]
	Modifications: HSV-1 IgG sandwich electrochemiluminescence immunoassays		85.9%/ 98.7%			[182]
	HSV-1 IgG multiplexed microparticle immunoassay		87.1%/ 98.2%			
	HSV-1 IgG chemiluminescent immunoassays		94.8%/ 90.4%			
	Additional methods: Avidity of anti-HSV-1 detection	Serum, plasma	100%/ 100%	Low avidity indicates an early stage of the disease; high avidity indicates an active infection		According to “HSV-1 IgG avidity-IMBIAN-ELISA” test
	Anti-HSV-1/2 immunoblotting	Serum, plasma	95%/ 100%	High sensitivity and specificity; confirms and clarifies ELISA results; detection of antibodies against certain clinically relevant viral antigens	Difficulty in interpreting results in some categories of patients; requires assessment by qualified specialists; expensive; labor-intensive and requires high qualifications of the researcher	[183]
Detection of viral antigen	Immunofluorescence assays in situ (IFA) Enzyme immunoassay (immunoperoxidase) assays (EIA) in the infected cells	Swabs	80–86%/ 98.3–100%	Rapid (<3 h) HSV-1 strains can be typed	Require fresh clinical samples; suboptimal sensitivity; requires high sample quality and high qualifications of the researcher	[184,185,186]
		Smears from urogenital lesions				
		Smear or vesicular fluid of exudate from base of vesicle				
		Corneal scrapings/corneal grafts				
Detection of virus by cell culture method	Clinical samples co-cultivation with the cultural cells	Swabs	80–90%/ 100%	Detection of infectious virus in a sample; high specificity Allows virus isolation	Require rapid transport, cooled, protected from light in virus transport medium; labor-intensive; expensive; specialized laboratories; long time to get results (4–7 days)	[187,188]
		Skin lesions				
		Vesicular fluid of exudate from base of vesicle				
		Mucosal sample without lesions				
		Conjunctival/corneal smears				
		Saliva				
		Urine				
		Blood				
		Biopsies				
	Modification: rapid culture method: co-cultivation of samples with sensitive cell culture for 24–48 h followed by IFA using monoclonal antibodies	The samples presented above	95%/ 100%	Faster than the classical culture method (24–48 h); confirmation of the HSV-1 presence in the clinical samples; determination of the number of viral infected cells per 10^5^ cells	Sample storage and transport conditions influence sensitivity; labor-intensive; expensive; specialized laboratories	[189]
Molecular-biological methods						
Detection of viral DNA and genes by PCR	HSV1 DNA detection and quantitation by classical or real-time PCR	Conjunctival/corneal smear	98%/ 100%	High sensitivity	Can be performed only in specialized laboratories	[187,188,190]
		Swabs		Currently “preferred” test (CDC 2010)	Not standardized	
		Skin lesions		Allows virus detection and typing in the same test	Not validated for all types of samples	
		Vesicular fluid or exudate from the base of the vesicle		Rapid (<3 h)	Risk of contamination (PCR)	
		Mucosal samples		May be automated	May be relatively expensive (real-time PCR)	
		Aqueous/vitreous humor		Labor efficient	Routine resistance genotyping not available	
		Cortico-spinal fluid				
		Blood and blood cells		Method of choice for cerebrospinal fluid (CSF)		
				Real-time PCR		
				Rapid amplification		
				Quantitative analysis		
				Method of choice for skin lesions		
				Reduced risk of contamination		
CRISPR/Cas-based diagnostics	MUSCA-PEC assay coupled with CRISPR/Cas12a for HSV-1 detection	Serum	96.2%/ 100%	High sensitivity and specificity	Not yet approved by the FDA	[191]
	LAMP-Cas12 diagnostic technology combined with gold nanoparticles	Tear specimens	93.9%/100%	High sensitivity and specificity; easy interpretation no equipment required (suitable for point-of-care detection of HSV-1)	Not yet approved by the FDA	[192]
	Warm-start rapid digital CRISPR approach (WS-RADICA)	Synthetic DNA fragments	99.6%/100%	High sensitivity and specificity	Not yet approved by the FDA	[193]
	Rapid isothermal CRISPR-Cas13a diagnostic test (HSV-SHERLOCK assay)	Clinical samples, swabs	96.2%/ 100%	High sensitivity and specificity; rapid	Not yet approved by the FDA; requires relatively expensive equipment (microplate reader)	[194]

## Data Availability

The datasets generated during and/or analyzed during the current study are available from the corresponding author on reasonable request.
